# Evaluation of Various Campylobacter-Specific Quantitative PCR (qPCR) Assays for Detection and Enumeration of Campylobacteraceae in Irrigation Water and Wastewater via a Miniaturized Most-Probable-Number–qPCR Assay

**DOI:** 10.1128/AEM.00077-16

**Published:** 2016-07-15

**Authors:** Graham S. Banting, Shannon Braithwaite, Candis Scott, Jinyong Kim, Byeonghwa Jeon, Nicholas Ashbolt, Norma Ruecker, Lisa Tymensen, Jollin Charest, Katarina Pintar, Sylvia Checkley, Norman F. Neumann

**Affiliations:** a School of Public Health, University of Alberta, Edmonton, Alberta, Canada; b Environmental Microbiology Program, Provincial Laboratory for Public Health, Edmonton, Alberta, Canada; c Water Quality Services, Calgary, Alberta, Canada; d Irrigation and Farm Water Division, Alberta Agriculture and Forestry, Lethbridge, Alberta, Canada; e Infectious Disease Prevention and Control Branch, Public Health Agency of Canada, Ottawa, Ontario, Canada; f Department of Ecosystem and Public Health, Faculty of Veterinary Medicine, University of Calgary, Calgary, Alberta, Canada; Wageningen University

## Abstract

Campylobacter spp. are the leading cause of bacterial gastroenteritis worldwide, and water is increasingly seen as a risk factor in transmission. Here we describe a most-probable-number (MPN)–quantitative PCR (qPCR) assay in which water samples are centrifuged and aliquoted into microtiter plates and the bacteria are enumerated by qPCR. We observed that commonly used Campylobacter molecular assays produced vastly different detection rates. In irrigation water samples, detection rates varied depending upon the PCR assay and culture method used, as follows: 0% by the de Boer Lv1-16S qPCR assay, 2.5% by the Van Dyke 16S and Jensen *glyA* qPCR assays, and 75% by the Linton 16S endpoint PCR when cultured at 37°C. Primer/probe specificity was the major confounder, with Arcobacter spp. routinely yielding false-positive results. The primers and PCR conditions described by Van Dyke et al. (M. I. Van Dyke, V. K. Morton, N. L. McLellan, and P. M. Huck, J Appl Microbiol 109:1053–1066, 2010, http://dx.doi.org/10.1111/j.1365-2672.2010.04730.x) proved to be the most sensitive and specific for Campylobacter detection in water. Campylobacter occurrence in irrigation water was found to be very low (<2 MPN/300 ml) when this Campylobacter-specific qPCR was used, with the most commonly detected species being C. jejuni, C. coli, and C. lari. Campylobacters in raw sewage were present at ∼10^2^/100 ml, with incubation at 42°C required for reducing microbial growth competition from arcobacters. Overall, when Campylobacter prevalence and/or concentration in water is reported using molecular methods, considerable validation is recommended when adapting methods largely developed for clinical applications. Furthermore, combining MPN methods with molecular biology-based detection algorithms allows for the detection and quantification of Campylobacter spp. in environmental samples and is potentially suited to quantitative microbial risk assessment for improved public health disease prevention related to food and water exposures.

**IMPORTANCE** The results of this study demonstrate the importance of assay validation upon data interpretation of environmental monitoring for Campylobacter when using molecular biology-based assays. Previous studies describing Campylobacter prevalence in Canada utilized primers that we have determined to be nonspecific due to their cross-amplification of Arcobacter spp. As such, Campylobacter prevalence may have been vastly overestimated in other studies. Additionally, the development of a quantitative assay described in this study will allow accurate determination of Campylobacter concentrations in environmental water samples, allowing more informed decisions to be made about water usage based on quantitative microbial risk assessment.

## INTRODUCTION

*C*ampylobacter spp. are Gram-negative, rod-shaped, motile bacteria of the class Epsilonproteobacteria and the family Campylobacteraceae, which contain the closely related genera Campylobacter, Arcobacter, and Helicobacter. It was only in the 1990s that Arcobacter and Campylobacter were proposed to be separate genera due to the observation that Arcobacter displays aerotolerance unlike Campylobacter, which requires a microaerophilic atmosphere for culture (1). Campylobacter and Arcobacter are present in the gut of warm-blooded animals such as birds, cattle, and pigs (2–6). Campylobacter is the leading cause of bacterial gastroenteritis in Canada, with an infection rate of 29/100,000 persons in 2013 (7). The predominant route of transmission is via contaminated foods (poultry in particular) (8), but with untreated water also being recognized as a potential source of infection. Campylobacteriosis rates in Canada have been shown to be elevated in comparison to those of control individuals if water consumption was from well water and the well was within 2 km of agricultural activity (9), and several campylobacteriosis outbreaks have been linked to water in Canada and Europe (10–13). Outbreaks are typically the result of either fecally contaminated surface runoff (manure) following rainfall or human sewage intrusion into source waters coupled with little or no treatment of drinking water supplies. High-intensity cattle, swine, or poultry farming combined with rainfall can result in fecal loading into groundwater and water of nearby waterways, which can subsequently be used for irrigation of crops. While there have been no confirmed campylobacteriosis outbreaks directly attributed to irrigation water, there have been multiple reports of gastroenteritis outbreaks associated with contaminated produce. Leafy and root vegetables have been associated with at least seven campylobacteriosis outbreaks between 1998 and 2008 in the United States (14). Additional studies have detected live Campylobacter spp. and closely related Arcobacter spp. on fresh produce (15, 16). Contamination of fresh produce with other enteric bacteria (Escherichia coli O157:H7) from water has led to gastroenteritis outbreaks (17), demonstrating the linkage between illness and consumption of contaminated vegetables. Direct and indirect costs due to gastroenteritis can be significant, e.g., >75 million euros/year in the Netherlands for campylobacteriosis alone in 2011 (18).

Understanding the influence of irrigation water quality on downstream risk, in terms of potential sources of contamination (i.e., wastewater or animal manure), is an important step in evaluating the public health risks posed by this organism in food and water and reducing the potential illness due to the consumption of contaminated produce. Detection and quantitation of Campylobacter typically involve a most-probable-number (MPN) culture enrichment step followed by a confirmation step, which may include PCR, plating on selective agar, and/or biochemical testing. Studies from Canada have reported Campylobacter from surface waters at high frequencies and concentrations (19–21). It is worth noting that few studies use the same PCR primers when molecular detection/confirmation of putative campylobacters is performed, making comparisons among different studies difficult. Additionally, due to the genetic similarity between Campylobacter and Arcobacter, great care must be taken to ensure the specificity of PCR assays. This is of paramount importance when dealing with environmental samples, as Arcobacter is likely to be numerically superior to Campylobacter due to its aerotolerance, ability to survive extended periods of time in water (22, 23), growth potential in certain water matrices (24), and ability to grow under the same culture conditions as Campylobacter (25).

When processing environmental water samples for Campylobacter enrichment, relatively large volumes are required. This necessitates a preprocessing step, typically filtration or centrifugation. Filtration may prove challenging or impractical with turbid water samples and is not fully compatible with the MPN format, as there is no consistent way to remove all cells from the filter before transfer to the multiple replicate wells required by the MPN format. Centrifugation has been shown to be highly effective at recovery of Campylobacter (26) and is directly compatible with the MPN format.

The enrichment and enumeration of Campylobacter spp. from poultry samples using an MPN format have been reported previously (27). However, the standard MPN format utilizes relatively large volumes and requires the use of a mixed-gas incubator to generate the microaerophilic atmosphere required to culture Campylobacter. A miniaturized MPN assay to quantitate Campylobacter bacteria from poultry samples has been described previously (28); it utilizes a microplate format allowing the use of small sealed chambers combined with microaerophilic atmosphere-generating sachets.

Here, our research examines the optimization of a miniaturized MPN-quantitative PCR (qPCR) assay that reduces the overall assay time to approximately 48 h in addition to using qPCR to “score” the MPN, ultimately leading to a more accurate enumeration of Campylobacter spp. This study also compares the performances of various Campylobacter qPCR assays in the context of surface and wastewater samples with respect to their specificity and sensitivity for detection of this microbe in environmental water samples.

## MATERIALS AND METHODS

### Bacterial strains and growth conditions.

Campylobacter type strains were purchased from Cedarlane Laboratories (Burlington, Ontario, Canada) for use in the development of the MPN-qPCR assay and included the following strains: (i) Campylobacter jejuni ATCC 29428; (ii) Campylobacter coli ATCC 33559; (iii) Campylobacter lari ATCC 35221; (iv) Campylobacter fetus ATCC 27374; (v) Campylobacter hyointestinalis ATCC 35217; and (vi) Campylobacter upsaliensis ATCC 43954. Isolates were initially cultured on blood agar plates (BAP) (Dalynn Biologicals, Calgary, Alberta, Canada) at 37°C in a Mitsubishi AnaeroPak system (ThermoFisher Scientific, Ottawa, Ontario, Canada) with MicroAero Paks generating a microaerophilic environment. Human-derived Campylobacter isolates were obtained from the Alberta Provincial Laboratory for Public Health (ProvLab) and were cultured as described for the type strains.

### Target cloning and limit-of-detection determination.

Campylobacter PCR targets were amplified from genomic DNA from the type strains (listed above) and cloned into pCR2.1-TOPO (ThermoFisher Scientific, Ottawa, Ontario, Canada) per the manufacturer's instructions. Clones were purified using the QIAprep spin kit per the manufacturer's instructions (Qiagen, Toronto, Ontario, Canada) and were sequenced by the Sanger method (Macrogen, Seoul, South Korea) to confirm identity. Clones were quantitated using the Qubit high-sensitivity DNA quantitation kit (ThermoFisher Scientific, Ottawa, Ontario, Canada). (q)PCR was run on serial dilutions of 50,000 to 0.5 copies per reaction as described below. The 95% confidence limit of detection (LOD_95_) was determined by the method of Wilrich and Wilrich (29) for each target assayed (Table 1).

**TABLE 1:** PCR assays used in this study and their limits of detection (LOD_95_)

Assay and primer	Oligonucleotide sequence (5′-3′)^*a*^	Targeted organism	Product size (bp)	Primer/probe concn (nM)^*b*^	Annealing temp (^o^C)	LOD_95_ copies (L/U)^*c*^	Reference
Linton 16S endpoint PCR							
Linton 16S F	GGATGACACTTTTCGGAGC	Campylobacter spp.	816	300/—	55	6.25 (1.5/17.1)	Linton et al. (32)
Linton 16S R	CATTGTAGCACGTGTGTC						
de Boer Lv1-16S qPCR							
Lv1 16S F	CCTGAMGCAGCAACGCC	Campylobacter spp.	107	300/200	60	3.2 (1.5/6.94)	de Boer et al. (30)
Lv1 16S R	CGGAGTTAGCCGGTGCTTATT						
Lv1 16S P	6-FAM-CTCCGAAAAGTGTCATCCT-NFQ-MGB						
Van Dyke 16S qPCR							
VD 16S F	CTGCTTAACACAAGTTGAGTAGG	Campylobacter spp.	287	300/100	60	43.4 (18.8/124.3)	Van Dyke et al. (19)
VD 16S R	TTCCTTAGGTACCGTCAGAA						
VD 16S P	6-FAM-CGCTCCGAAAAGTGTCATCCTCC-BHQ1						
Jensen *glyA* qPCR							
glyA cc F	GTTGGAGCTTATCTTTTTGCAGACA	Campylobacter coli	80	300/100	60	2.1 (0.5/8.4)	Jensen et al. (31)
glyA cc R	TGAGGAAATGGACTTGGATGCT						
glyA cc P	VIC-TGCTACAACAAGTCCAGCAATGTGTGCA-TAMRA						
glyA cj F	TAATGTTCAGCCTAATTCAGGTTCTC	Campylobacter jejuni	135	300/100	60	2.1 (0.5/8.4)	Jensen et al. (31)
glyA cj R	GAAGAACTTACTTTTGCACCATGAGT						
glyA cj P	6-FAM-AATCAAAGCCGCATAAACACCTTGATTAGC-TAMRA						
glyA cl F	CAGGCTTGGTTGTAGCAGGTG	Campylobacter lari	96	300/100	60	2.1 (0.5/8.4)	Jensen et al. (31)
glyA cl R	ACCCCTTGGACCTCTTAAAGTTTT						
glyA cl P	VIC-CATCCTAGTCCATTCCCTTATGCTCATGTT-TAMRA						
Yamazaki multiplex PCR							
C.coli ask F	GGTATGATTTCTACAAAGCGAG	Campylobacter coli	502	200/—	58	6.25 (2.3/17.1)	Yamazaki-Matsune et al. (34)
C.coli ask R	ATAAAAGACTATCGTCGCGTG						
C.fet cstA F	GGTAGCCGCAGCTGCTAAGAT	Campylobacter fetus	359	200/—	58	6.25 (2.3/17.1)	Yamazaki-Matsune et al. (34)
C.fet cstA R	AGCCAGTAACGCATATTATAGTAG						
C.lari glyA F	TAGAGAGATAGCAAAAGAGA	Campylobacter lari	251	200/—	58	18.9 (6.1/59.0)	Yamazaki-Matsune et al. (34)
C.lari glyA R	TACACATAATAATCCCACCC						
C.jej 0414 F	CAAATAAAGTTAGAGGTAGAATGT	Campylobacter jejuni	161	200/—	58	30.4 (9.6/95.8)	Yamazaki-Matsune et al. (34)
C.jej 0414 R	CCATAAGCACTAGCTAGCTGAT						
C.ups lpxA F	CGATGATGTGCAAATTGAAGC	Campylobacter upsaliensis	86	200/—	58	11.5 (4.1/32.3)	Yamazaki-Matsune et al. (34)
C.ups lpxA R	TTCTAGCCCCTTGCTTGATG						
Khan-ITS							
ICC-F	GAAGTATCAATCTTAAAAAGATAA	Campylobacter coli	72	300/—	46	64.8 (21.4/196.0)	Khan et al. (35)
ICC-R	AAATATATACTTGCTTTAGATT						
ICJ-F	CTTAGATTTATTTTTATCTTTAACT	Campylobacter jejuni	349			64.8 (21.4/196.0)	
ICJ-R	ACTAAATGATTTAGTCTCA						
ICL-F	CTTACTTTAGGTTTTAAGACC	Campylobacter lari	279			64.8 (21.4/196.0)	
ICL-R	CAATAAAACCTTACTATCTC						
*hsp60* qPCR							
hsp60 F	CTCTTCATTAAAAGAGATGTTACCAATTTT	Arcobacter butzleri	91	300/100	60	ND	de Boer et al. (30)
hsp60 R	CACCATCTACATCTTCWGCAATAATTACT						
hsp60 P	6-FAM-CTTCCTGATTGATTTACTGATT-NFQ-MGB						
IAC							
IAC F	CTAACCTTCGTGATGAGCAATCG	NA^*d*^	198	400/100	60	4.3 (2.3/8.1)	Deer et al. (33)
IAC R	GATCAGCTACGTGAGGTCCTAC						
IAC P	VIC-AGCTAGTCGATGCACTCCAGTCCTCCT-NFQ-MGB						

a 6-FAM, 6-carboxyfluorescein; BHQ1, black hole quencher 1; TAMRA, 6-carboxytetramethylrhodamine.

b —, no probe (endpoint PCR only).

c LOD_95_, 95% confidence limit of detection; L, lower confidence interval; U, upper confidence interval; ND, not determined.

d NA, not applicable.

### Sample preparation for irrigation water.

**FIG 1:**
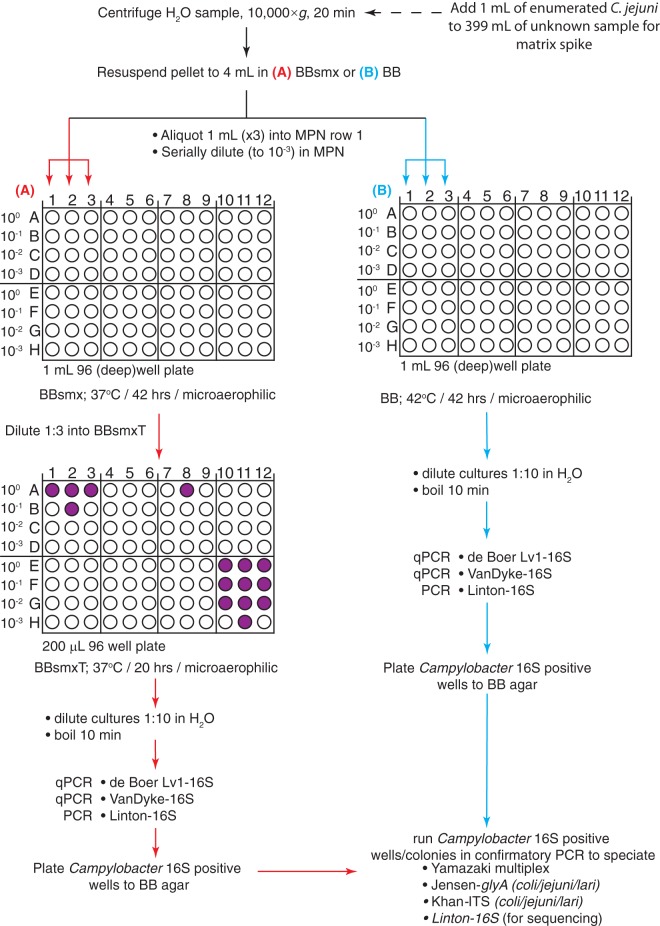
Campylobacter MPN-qPCR assays designed for use with water samples. Two assay variations were designed: (A) a double-enrichment MPN assay with culture in Bolton broth containing selective supplements, sulfamethoxazole (BBsmx), and the metabolic indicator TTC (second plate only; BBsmxT) at 37°C, as modified from the method of Chenu et al. (28); and (B) a single-enrichment MPN assay without metabolic indicator, with culture in Bolton broth plus selective supplements only (BB) at 42°C. Campylobacter-specific (q)PCR performed on MPN cultures determined which wells to plate onto solid agar (BB), followed by a confirmatory PCR for identification to the species level.

Irrigation water samples from southern Alberta, collected during the 2014 and 2015 field seasons, were sampled in 1-liter sterile plastic jars and shipped overnight on ice to the ProvLab in Edmonton, Alberta, Canada. The following morning, 400 ml of each sample was spun at 10,000 × *g* in sterile Nalgene bottles in a Sorvall RC5C centrifuge at 20°C for 20 min (Fig. 1). For the 2014 samples, the resulting pellets were resuspended in Bolton broth (BB) (Oxoid CM0983; ThermoFisher, Nepean, Ontario, Canada) containing Bolton selective supplements (Oxoid R0183) and 25 mg/liter sulfamethoxazole (BBsmx) (Sigma, Markham, Ontario, Canada) to a total volume of 4 ml. For the 2015 samples, sulfamethoxazole was omitted.

### Sample preparation for wastewater.

Sewage samples were provided by the City of Calgary from the Pine Creek wastewater treatment plant, a system that serves a mostly residential portion of the city. Samples were collected from the raw, post-grit-screened influent and shipped on ice by courier to the ProvLab in Edmonton within 24 h of collection. Samples were processed as described for irrigation water, with the following slight modifications: 100 ml of wastewater was diluted to 400 ml with sterile buffered water prior to centrifugation. Resuspension of the resulting pellet was done either in Bolton broth with selective supplement (BB) medium or BB supplemented with rifampin (10 mg/liter; Sigma, Markham, Ontario, Canada) and polymyxin B (5,000 IU/liter; Sigma, Markham, Ontario, Canada) (BBRP).

### Matrix spikes.

In order to validate assay recoveries, matrix spikes were performed on a random irrigation water sample from the sampling date under interrogation. C. jejuni ATCC 29428 was cultured as described above, scraped from the BAP agar into 10 ml of phosphate-buffered saline, and enumerated by plating serial dilutions back to BAP. One-milliliter-volume dilutions in the 10^−4^ to 10^−6^ range of these pure cultures were spiked into a random water sample prior to centrifugation and processed as described for the other samples. Spikes ranged from 10^0^ to 10^5^ CFU per sample. The number of C. jejuni bacteria spiked in each sample varied by week due to the fact that the bacteria in the spiked sample were enumerated concomitantly with those in the cultured stock in order to ensure accurate quantitation. This was due to our concern that the viability of Campylobacter could be affected in stock cultures when they were stored at 4°C for 48 h while plate quantifications were performed.

### Miniaturized MPN-qPCR assay for irrigation water.

The miniaturized three-tube MPN assay was based on methods described by Chenu et al. (28) with minor modifications. The resuspended pellet was split into three 1-ml aliquots and added to an initial deep-well (1-ml, 96-well) MPN plate (Greiner BioOne), followed by serial dilution to 10^−3^ (for irrigation water) in BB (2015 samples) or BBsmx (2014 samples) media. The plates were covered with loose-fitting hard-shell lids and placed in AnaeroPack jars (Mitsubishi) with AnaeroPack-MicroAero (Mitsubishi) and incubated for 42 to 44 h at 37°C (2014 samples) or 42°C (2015 samples). Initially, incubation at 37°C was chosen based on the findings of Khan et al. (20), who observed that incubation at 37°C led to an increased overall recovery and a greater diversity of Campylobacter spp. from water. After incubation, the plates were opened and diluted 1:3 into a second MPN plate (0.2 ml, 96 wells; Greiner BioOne) containing BBsmx plus 150 μg/ml of the metabolic indicator triphenyltetrazolium chloride (TTC; Sigma, Markham, Ontario, Canada) (BBsmxT). Unlike the method of Chenu et al. (28), agar was not added to this medium and it was left as a liquid broth. This second plate was incubated as for the first plate but only for 18 to 20 h at 37°C. In the 2015 samples, the second plate was eliminated completely and the incubation temperature was changed to 42°C (based on results using wastewater as a matrix), in order to compare the relative sensitivities of a single-step enrichment assay for screening samples.

### Miniaturized MPN-qPCR assay for wastewater.

Due to the fact that very few irrigation water samples were contaminated with Campylobacter in Alberta, we used wastewater as a matrix to further optimize detection. However, because of the high microbial content of wastewater (and, in particular, Arcobacter spp.), we examined the effects of increasing the stringency of the culture conditions in some experiments by raising the incubation temperature to 42°C and adding two additional antibiotics (rifampin and polymyxin B). MPN cultures were grown at 37°C and 42°C in either BB or BBRP medium. Additionally, MPN cultures were diluted to 10^−7^ to more accurately enumerate high-prevalence contaminating species (Arcobacter spp.). Only a single enrichment step was performed; time, temperature, and atmosphere conditions for this primary enrichment step were the same as for irrigation water samples.

### (q)PCR.

After incubation in the primary or secondary MPN plates, cultures were diluted 1:10 in H_2_O and heated at 95°C for 10 min to lyse the cells. The initial screening for Campylobacter presence was performed on an Applied Biosystems TaqMan 7500 fast real-time PCR system using the qPCR primer/probe conditions described by de Boer et al. (30) (here referred to as the de Boer Lv1-16S qPCR assay) (Table 1) using 5 μl of the diluted, boiled cultures as the template. Subsequently, screening was performed with additional Campylobacter (q)PCR assays to compare the specificities of the primers for the detection of Campylobacter. These included the PCR primers and conditions described by Van Dyke et al. (19) (Van Dyke 16S qPCR), Jensen et al. (31) (Jensen *glyA* qPCR), and Linton et al. (32) (Linton 16S endpoint PCR), as outlined in Table 1. All qPCR assays were fast cycled using 1× TaqMan fast advanced master mix (ThermoFisher Scientific, Ottawa, Ontario, Canada) and 200 μg/ml bovine serum albumin (BSA; Sigma, Markham, Ontario, Canada) with the following cycling conditions: 50°C for 2 min, followed by 95°C for 20 s and then 40 cycles of 95°C for 3 s and 60°C for 30 s. The de Boer Lv1-16S and Van Dyke 16S assays were duplexed with an internal amplification control (IAC) assay (Table 1) in which 100 copies of IAC plasmid (33) was spiked into each reaction mixture to determine if PCR inhibition was occurring. Samples were deemed inhibited if cycle threshold (*C_T_*) values of the IAC assay were shifted by ≥3. High-performance liquid chromatography-purified primers and probes were purchased from ThermoFisher Scientific. Primer/probe concentrations for each assay are shown in Table 1.

The Linton 16S endpoint PCR was also performed on all 2014 irrigation water MPN cultures. These reactions were amplified using Maxima hot start master mix (ThermoFisher Scientific, Ottawa, Ontario, Canada) containing 200 μg/ml BSA using the following cycling conditions: 95°C for 4 min, followed by 35 cycles of 95°C for 20 s, 55°C for 30 s, and 72°C for 1 min. Reactions were run on 2% agarose gels and photographed on an ImageQuant LAS4000 imager (GE Biosciences, Mississauga, Ontario, Canada).

MPN cultures were also screened with an Arcobacter butzleri-specific qPCR assay targeting the heat shock protein 60 gene (*hsp60*), as described by de Boer et al. (30) (Table 1). This was undertaken to resolve the specificity of the Campylobacter assays as a result of significant Arcobacter growth in the MPN cultures that confounded Campylobacter identification. Cycling conditions were as described for the Campylobacter qPCR assays described above.

### Species confirmation.

MPN wells that showed exponential amplification by the Campylobacter genus-specific qPCR were selected for secondary screening for confirmation of the occurrence of Campylobacter spp. by using the methods of Yamazaki-Matsune et al. (34) (here referred to as the Yamazaki multiplex PCR; Table 1). As the Yamazaki multiplex PCR assay did not always identify a putative Campylobacter species, the Linton 16S PCR amplicons were also sequenced by Sanger method sequencing (Macrogen, Seoul, South Korea), and the resulting DNA sequences were subjected to Basic Local Alignment Search Tool (BLAST) analysis to confirm identity. qPCR-positive wells were plated on Bolton agar, and putative Campylobacter colonies were enriched in Bolton broth overnight at 37°C, followed by PCR (described below) to confirm identity.

For samples from the 2015 irrigation season, Campylobacter-positive samples and isolates were also screened by the Jensen *glyA* qPCR (three assays) (Table 1) and the Khan ITS multiplex PCR (35) (Table 1) to identify Campylobacter bacteria to the species level. The Jensen *glyA* assays for C. jejuni and C. lari were performed together (i.e., duplex), and the C. coli assay was processed by itself (i.e., simplex). Cycling conditions were the same as for the other assays described above. The Khan ITS assay was run with Qiagen multiplex PCR master mix (Qiagen, Toronto, Ontario, Canada) with the addition of 200 μg/ml BSA per the manufacturer's instructions. Reactions were cycled using the following program: 95°C for 15 min, followed by 35 cycles of 95°C for 30 s, 46°C for 90 s, and 72°C for 60 s. Reaction mixtures were run on 2.25% agarose gels and photographed as described above. Campylobacter isolates from human feces and wastewater were tested with the same PCR panel described above.

### MPN enumeration.

The bacteria in the MPN cultures were enumerated based on either the TTC metabolic indicator change after selective enrichment as described by Chenu et al. (28) and/or the Campylobacter qPCR results. Wells were deemed positive by qPCR if they displayed exponential amplification with a *C_T_* value of <35 and with no inhibition detected in IAC controls. Standard three-tube MPN tables were followed to determine an MPN/300 ml (irrigation water) or an MPN/100 ml (wastewater).

## RESULTS

### Campylobacter MPN-qPCR method validation using matrix spikes.

In order to validate the performance of the MPN-qPCR assay, C. jejuni ATCC 29428 was used as a matrix spike in autoclaved irrigation water on each of the 16 sampling dates in the 2014 and 2015 irrigation field seasons. The initial spikes prior to the centrifugation step ranged from ∼10^0^ to 10^5^ CFU in each sample. In 15 of 16 cases, the matrix spike was observed by qPCR positivity (Van Dyke 16S assay) in the MPN cultured wells (Table 2). Accounting for serial dilution in the MPN plates, PCR positivity was observed across the spiked cultures, with an inoculum equivalency between 1 and 500 CFU being detected by the method after culture enrichment. Matrix spike recoveries were calculated based on the calculated MPN of the spike (based on qPCR positivity of the individual wells) versus the plate-counted inoculum and ranged from 0.5 to 71% in the 37°C culture method and 9 to 171% in the 42°C culture method (assay development described below). Matrix spike recoveries were consistently higher in the 42°C method, and this was attributed to the reduction in competition from Arcobacter and other bacteria (expanded upon in “Campylobacter detection in wastewater” below). The A. butzleri MPN in each spike sample was generally lower in the 2015 samples cultured at 42°C than in the 2014 samples cultured at 37°C (Table 2), supporting this hypothesis. These matrix spike results confirm the sensitivity of both the centrifugation and culture/PCR portions of the assay, with as little as 1 CFU of C. jejuni detectable by the method after culture enrichment (Table 2).

**TABLE 2:** Matrix spike recoveries of C. jejuni in irrigation water using an MPN-qPCR assay

Sample	No. of C. jejuni CFU/spike^*a*^	MPN detection limit (no. of C. jejuni CFU)	Spike recovery of C. jejuni (%)	A. butzleri MPN in spike sample^*g*^
1^*b*^	1.1 × 10^1^	8.0 × 10^0^	11.3	0
2^*b*^	3.9 × 10^2^	1.0 × 10^0^	71.2	0
3^*b*^	5.9 × 10^5^	4.4 × 10^2^	>0.5	9.3
4^*b*^	4.0 × 10^4^	3.0 × 10^3^	0.7	46
5^*b*^	5.8 × 10^4^	4.4 × 10^1^	>14.6	0.4
6^*b*^	2.4 × 10^3^	2.0 × 10^0^	11	>2,400
7^*b*^	3.8 × 10^2^	2.9 × 10^2^	0.1	111
8^*b*^	1.2 × 10^4^	9.0 × 10^0^	10.1	46
9^*c*^	2.6 × 10^0^	0.8 × 10^0^	15.4	0
10^*c*^	IND^*d*^	DNQ^*e*^	DNQ	0
11^*c*^	1.0 × 10^2^	2.5 × 10^0^	9.3	2.3
12^*c*^	ND^*f*^	ND	ND	0
13^*c*^	2.6 × 10^2^	0.6 × 10^0^	177	0.4
14^*c*^	2.8 × 10^2^	0.7 × 10^0^	33.2	0.9
15^*c*^	2.8 × 10^2^	0.7 × 10^0^	164.3	0
16^*c*^	1.4 × 10^2^	0.4 × 10^0^	171.4	0

a Spiked into 400 ml of irrigation water prior to centrifugation step.

b Double-enrichment MPN assay at 37°C (2014 samples).

c Single-enrichment MPN assay at 42°C (2015 samples).

d IND, indeterminate. Could not enumerate cells before spike due to swarming on plate.

e DNQ, detected by qPCR, but enumeration not possible due to swarming on plate.

f ND, not detected. No cells went into spike.

g Determined by the de Boer *hsp60* qPCR

### Campylobacter occurrence in irrigation water in 2014.

The initial development of the MPN-qPCR assay was performed using 80 irrigation water samples from southern Alberta between June and September 2014. For these samples, a double-enrichment MPN assay at 37°C was performed with the metabolic indicator TTC in the second enrichment (Fig. 1A and B), as described by Chenu et al. (28). Initial qPCR screening with the Campylobacter genus-specific de Boer Lv1-16S qPCR assay (Table 1) failed to yield any samples showing exponential qPCR amplification (Table 3), yet 89% of the samples displayed a color change in the medium, indicating bacterial growth in these wells. In these metabolism-positive samples (i.e., TTC positive), an average MPN of 94.2 ± 178/300 ml was observed, which is an underestimation of occurrence, since four samples were not included in the calculation as they were outside the dynamic range of the MPN assay (>2,400 MPN/300 ml). Based on reports of high levels of Campylobacter detected in other Canadian studies, we rescreened the MPN cultures using the Linton 16S endpoint assay as described by Khan et al. (20, 21) (Table 1). The Linton 16S assay yielded a 75% positivity rate across MPN wells, with an average MPN value of 55.7 ± 184/300 ml (Table 3), with one sample excluded from the calculation for having an MPN value of >2,400/300 ml. To confirm sequence identity and specificity of the assay, 65 of the Linton 16S endpoint PCR amplicons (originating from 60 water samples) were sequenced and subjected to BLAST analysis. DNA sequence analysis revealed that 60 of 65 amplicons were actually Arcobacter butzleri, while 3 of 65 were confirmed to be Arcobacter cryaerophilus. Only 2 of 65 were confirmed as C. lari, suggesting that the primer set of Linton et al. (32) was not specific to the Campylobacter genus. These results made us question the Campylobacter PCR assay specificities used by other researchers when examining the occurrence of Campylobacter in water. As such, all MPN cultures were rescreened using five additional PCR assays (see below).

**TABLE 3:** Frequency and enumeration of Campylobacter and Arcobacter bacteria in irrigation water samples from 2014 as detected by a double-enrichment MPN-(q)PCR assay at 37°C (*n* = 80)

Bacterium and assay	No. (%) of bacteria detected	MPN (avg ± SD)/300 ml^*a*^
Campylobacter		
Linton 16S endpoint PCR	60 (75)	55.7^*b*^ ± 184
de Boer Lv1-16S qPCR	0 (0)	0
Van Dyke 16S qPCR	2 (2.5)	<1
Jensen *glyA* qPCR	2 (2.5)	<1
Arcobacter		
*hsp60* qPCR	63 (79)	96.8^*c*^ ± 257
Metabolism (TTC)	71 (89)	94.2^*d*^ ± 178

a Calculated only on samples within the dynamic range of the assay (0 to 2,400 MPN).

b One sample removed from calculation due to a value of >2,400/300 ml obtained in MPN assay.

c Two samples removed from calculation due to values of >2,400/300 ml obtained in MPN assay.

d Four samples removed from calculation due to values of >2,400/300 ml obtained in MPN assay.

### Campylobacter PCR assay sensitivity/specificity comparisons.

Due to the lack of concordance between the de Boer Lv1-16S qPCR, Linton 16S endpoint PCR, and metabolic TTC assays, we rescreened all 2014 irrigation samples from MPN cultures by using the Van Dyke 16S qPCR, the Jensen *glyA* assays (C. coli/C. jejuni/C. lari), and an A. butzleri-specific qPCR assay (*hsp60*) (Table 1). The Van Dyke 16S qPCR and Jensen *glyA* qPCR assays both identified the two samples previously classified as C. lari through sequencing of the Linton 16S PCR amplicons, both of which were missed by the de Boer Lv1-16S qPCR assay (Table 3). The Van Dyke 16S qPCR and Jensen *glyA* qPCR assays did not identify any additional Campylobacter-positive samples not identified by Linton 16S amplicon sequencing, demonstrating their strong specificity to the Campylobacter genus (and not Arcobacter).

**FIG 2:**
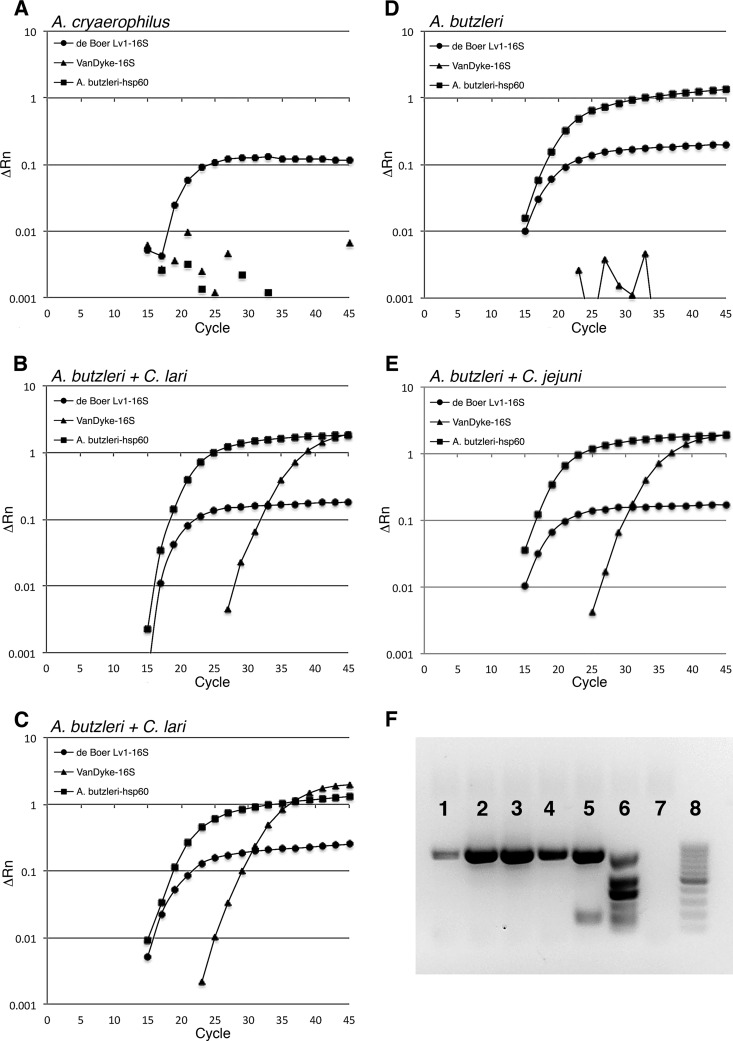
Five MPN cultures amplified by various qPCR assays for Campylobacter (de Boer Lv1-16S, circles; Van Dyke 16S, triangles) and Arcobacter butzleri (*hsp60*; squares). The five wells were determined to contain Arcobacter cryaerophilus (A), A. butzleri and C. lari (B and C), A. butzleri (D), and A. butzleri with a C. jejuni spike (E). (F) The same samples were run with the Yamazaki multiplex PCR (containing the Linton 16S assay amplicon), with individual lanes 1 to 5 corresponding to panels A to E. Lane 6, Yamazaki multiplex positive control; lane 7, no-template control; lane 8, 100-bp ladder. The Van Dyke 16S assay was able to amplify Campylobacter in the presence of Arcobacter, while the de Boer Lv1-16S assay did not, due to a cross-reaction with Arcobacter. The Yamazaki multiplex PCR was unable to identify the two samples containing C. lari (B and C) but could identify C. jejuni from a matrix spike sample (E). ΔRn, normalized fluorescence minus the background fluorescence, where normalized fluorescence refers to the ratio of the probe fluorescence to the fluorescence of the passive reference dye (ROX).

During the initial PCR screen with the de Boer Lv1-16S assay, nonexponential amplification was often observed (Fig. 2) in a portion of the irrigation water samples, which we originally assumed to be nonspecific. However, this nonspecific amplification often coincided with the presence of A. butzleri in the sample, as determined by the *hsp60* amplification assay results (Fig. 2B to E). In some cases, low-level nonspecific amplification was observed in the de Boer Lv1-16S assay in the absence of a positive A. butzleri *hsp60* assay result (Fig. 2A). In these cases, sequencing of the Linton 16S amplicon determined that A. cryaerophilus was present, suggesting that the Linton 16S endpoint PCR assay cross-reacted with the 16S gene of both A. butzleri and A. cryaerophilus while the Arcobacter hsp60 assay did not recognize A. cryaerophilus. Interestingly, in situations where both Arcobacter and Campylobacter were observed growing in the same well, not all assays were able to detect Campylobacter against this Arcobacter background. This may be due to the fact that based on MPN values, Arcobacter spp. were numerically superior (2 to 3 log_10_) to Campylobacter spp. and that PCR bias may occur, especially for assays that display some weak cross-amplification between the two genera. For MPN wells in which A. butzleri and C. lari were present (Fig. 2B and C), or for which A. butzleri and C. jejuni (Fig. 2E) were present, the *hsp60* assay was positive for A. butzleri and the Van Dyke 16S assay was positive for Campylobacter, but the de Boer Lv1-16S assay displayed nonexponential amplification curves indicative of Arcobacter. This result suggests that PCR bias may occur in the de Boer Lv1-16S assay and therefore it may not be useful for detection of Campylobacter from samples in which Arcobacter may be present.

**FIG 3:**
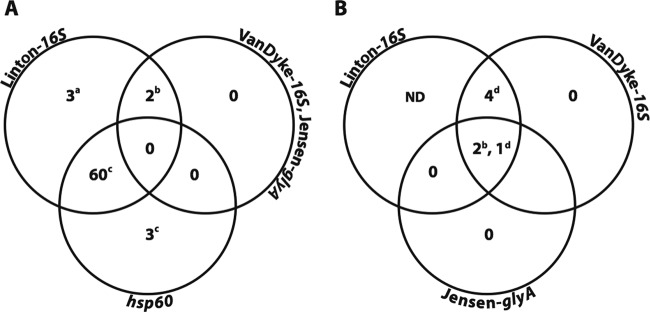
Venn diagram depicting primer specificity of various Campylobacter and Arcobacter (q)PCR assays on enriched MPN cultures from irrigation water in 2014 (A) and 2015 (B). In 2015, six samples were positive for Campylobacter, with one sample containing two different species. The Linton 16S and Van Dyke 16S assays are both reported to target Campylobacter spp. (i.e., are genus specific), while the Jensen *glyA* assay represents three individual assays to target C. coli/C. jejuni/C. lari (i.e., are species specific). The *hsp60* assay is designed to target Arcobacter butzleri only. The 16S gene sequence results confirmed the following species identifications, as indicated by superscript lowercase letters in the figure: a, Arcobacter cryaerophilus; b, Campylobacter lari; c, Arcobacter butzleri; d, Campylobacter jejuni/C. coli (not polymorphic enough to resolve). ND, not determined.

Overall, A. butzleri was detected in 79% of samples and on a well-to-well basis closely mimicked the Linton 16S assay results described above (Table 3), suggesting that A. butzleri was the major contaminating bacterial species in the MPN cultures and present in irrigation water at an MPN of 55.7 ± 184/300 ml (based on the Linton 16S assay) to 96.8 ± 257/300 ml (based on the *hsp60* assay). The MPN associated with the Linton 16S assay is slightly lower than those from assays using *hsp60* and TTC, likely due to the fact that amplification of Arcobacter by the Linton primers is a nonspecific reaction with low PCR efficiency. These MPNs are all likely an underestimate of the true occurrence of Arcobacter, since two samples were excluded from the calculation because the MPN exceeded the upper limit of detection of 2,400/300 ml. When these same samples were amplified by the Yamazaki multiplex PCR, the 16S band (same as that for the Linton 16S assay) was positive for all samples, albeit with differing amplification efficiencies (Fig. 2F). The Yamazaki multiplex PCR was unable to detect the two samples containing C. lari (Fig. 2F, wells 2 and 3) but could detect the C. jejuni from the matrix spike (Fig. 2F, well 5). Based on the results from all PCR assays tested, only the Van Dyke 16S qPCR-positive samples were confirmed to be Campylobacter positive (Fig. 3A), suggesting that the Van Dyke 16S assay was the most specific and accurate assay for use in screening MPN cultures for the presence of Campylobacter, despite the fact that the LOD_95_ for this assay was at least an order of magnitude higher than that of the de Boer Lv1-16S assay (Table 1). All subsequent samples (2015 irrigation samples and wastewater) were screened using the Van Dyke 16S assay.

### MPN enumeration.

For determination of the MPN of Campylobacter spp. growing in the enrichment broth, we compared two methods: PCR positivity in the culture well and color change in the metabolic indicator, TTC, as described by Chenu et al. (28). TTC color change resulted in the identification of 89% of the 2014 irrigation water samples as positive for metabolism in Bolton selective enrichment broth when cultured at 37°C, with an average MPN value of 94.2 ± 178/300 ml (Table 3). The MPN values based on TTC ranged from 0 to >2,400/300 ml, the limit of our assay for irrigation water. When PCR positivity was used to determine MPN values, the results differed based on the PCR assay used (see above). The Linton 16S assay resulted in a positivity rate of 75% with an average MPN of 55.7 ± 184/300 ml (range, 0 to >2,400), whereas both the Van Dyke 16S and Jensen *glyA* assays resulted in a positivity rate of 2.5% with an average MPN of <1/300 ml (Table 3). When the same cultures were enumerated for Arcobacter butzleri (*hsp60* assay), a positivity rate of 79% was observed, with an average MPN of 96.8 ± 257/300 ml (range, 0 to >2,400). The majority of wells positive for metabolism (TTC) were also positive for A. butzleri by the *hsp60* assay, suggesting that this species was the major nontarget microbe growing in the Bolton broth culture medium and metabolizing the TTC.

### Campylobacter detection in wastewater.

Due to the relatively low prevalence and concentration of Campylobacter spp. in irrigation water samples in Alberta, we adapted the Campylobacter MPN-qPCR for use with raw sewage in order to further optimize the MPN-qPCR assay. However, due to the high bacterial content in this water matrix, the input volume of the assay was reduced to 100 ml and a 42°C incubation temperature was utilized to suppress growth of competing microbiota. Sulfamethoxazole was also removed from the Bolton broth, since preliminary testing determined that it provided no reduction in growth from competing bacteria (data not shown), likely due to this drug being commonly found in wastewater effluent (36). We tested two temperature conditions (37°C versus 42°C) in combination with two antibiotic combinations (Bolton broth with selective supplement [BB] versus BB plus rifampin and polymyxin B [BBRP]) in a single-enrichment MPN-qPCR (Fig. 1B) for Campylobacter. Each of the four culture combinations were tested with three Campylobacter (q)PCR assays (de Boer Lv1-16S qPCR, Van Dyke 16S qPCR, Linton 16S endpoint PCR) and one A. butzleri qPCR assay (*hsp60*).

Wastewater proved a very challenging matrix for the recovery of Campylobacter growth in the MPN assay. Frequently, Campylobacter could not be detected in the undiluted wastewater MPN samples, an outcome attributed to the intense microbial growth competition in wastewater (i.e., Arcobacter) and not PCR inhibition (based on IAC reactions). Arcobacter was present in wastewater at concentrations up to 4 log_10_ higher than Campylobacter (Table 4), suggesting that it (and potentially other bacterial species) simply outcompetes the Campylobacter spp. for available resources when seeded at high concentrations.

**TABLE 4:** Enumeration of Campylobacter and Arcobacter bacteria in raw sewage (2 trials) as detected by an MPN-qPCR single-enrichment assay with four culture conditions and five different PCR assays

Bacterium and PCR assay	Trial	Value under indicated culture conditions^*a*^							
		BB, 37°C		BB, 42°C		BBRP, 37°C		BBRP, 42°C	
		MPN/100 ml	Avg *C_T_*	MPN/100 ml	Avg *C_T_*	MPN/100 ml	Avg *C_T_*	MPN/100 ml	Avg *C_T_*
Campylobacter									
de Boer Lv1-16S qPCR	1	0	NA^*c*^	4.3 × 10^2^	19.6	6.2 × 10^0^	19.7	2.4 × 10^2^	16.4
Van Dyke 16S qPCR	1	3.6 × 10^0^	42.9	4.6 × 10^2^	26.8	9.2 × 10^1^	34.3	2.3 × 10^2^	21.5
Jensen *glyA* qPCR	1	3.0 × 10^0^	37.1	4.6 × 10^2^	25.9	9.3 × 10^1^	30.8	2.4 × 10^2^	20.2
Linton 16S endpoint PCR	1	1.1 × 10^6^	NA	4.6 × 10^2^	NA	1.1 × 10^2^	NA	2.4 × 10^2^	NA
de Boer Lv1-16S qPCR	2	0	NA	1.5 × 10^2^	21.9	9.4 × 10^0^	17.9	9.3 × 10^1^	18.5
Van Dyke 16S qPCR	2	2.0 × 10^1^	36.7	4.6 × 10^2^	29.7	4.3 × 10^2^	27.4	9.3 × 10^1^	23.0
Jensen *glyA* qPCR	2	3.0 × 10^2^	24.9	4.3 × 10^2^	28.6	4.3 × 10^2^	27.0	1.5 × 10^2^	26.2
Linton 16S endpoint PCR	2	1.5 × 10^6^	NA	4.6 × 10^2^	NA	4.6 × 10^2^	NA	1.1 × 10^2^	NA
Arcobacter hsp*60* qPCR	1	2.4 × 10^5^	18.4	7.5 × 10^4^	35.1	2.4 × 10^3^	30.2	2.4 × 10^3^	37.9
	2	2.4 × 10^5^	19.6	9.3 × 10^3^	35.5	1.3 × 10^3^	29.0	2.1 × 10^2^	35.8

a BB, Bolton broth; BBRP, Bolton broth plus rifampin and polymyxin B.

c NA, not applicable.

The addition of rifampin and polymyxin B to Bolton broth increased the suppression of background microbiota in wastewater, with a concomitant reduction in *C_T_* values in the Campylobacter qPCR assays (Table 4), suggesting a reduction in growth competition from Arcobacter and other bacteria and an increase in the overall growth of Campylobacter spp. in these cultures. A. butzleri levels were reduced by ∼2 log_10_ in cultures containing the two additional antibiotics (Table 4). The lowering of *C_T_* values in the Campylobacter assays in BBRP medium, however, came at a cost of lower MPN values (∼40% lower in BBRP at 42°C than in BB at 42°C). While the Campylobacter MPNs were not substantively different between the three different qPCR assays tested, the *C_T_* values for the de Boer Lv1-16S assay were significantly lower (6 to 15 *C_T_* units lower). This was attributed to nonspecific amplification, with the high level of Arcobacter bacteria present affecting overall fluorescence in the qPCR.

Culturing at 42°C had the benefit of reducing the overall A. butzleri levels by ∼1 log_10_ and concomitantly raising the *C_T_* values (19.0 for BB at 37°C versus 35.3 for BB at 42°C), suggesting that A. butzleri grew poorly in BB at 42°C (Table 4). In BBRP medium, however, another phenomenon was observed. While A. butzleri MPN values were 1 to 2 log_10_ lower in BBRP than in BB at 37°C, they were the same in BBRP at 37°C and 42°C (Table 4). This suggests the presence of multiple strains of A. butzleri, with one or more of these strains being inherently resistant to all six antimicrobials present in the BBRP medium and tolerant to 42°C, albeit with a lower growth rate.

The average Campylobacter MPNs were not significantly different between the BB and BBRP cultures at 42°C, while culture at 37°C allowed too much background growth for Campylobacter to survive, as evidenced by the low MPN values observed (Table 4). Based on the growth characteristics of Campylobacter and Arcobacter under the four culture conditions tested, we achieved the best compromise for encouraging Campylobacter growth and suppressing Arcobacter by culture in standard Bolton broth with selective supplements at 42°C. This methodology allowed for optimal recovery of thermotolerant Campylobacter spp. in a complex wastewater matrix and was subsequently applied to irrigation water samples for the 2015 field season.

### Campylobacter detection in irrigation water in 2015.

Following the optimization of the MPN-qPCR assay for irrigation and wastewater samples, an additional set of irrigation water samples (*n* = 74) was tested in 2015. Based on optimization from wastewater samples, a single-enrichment MPN assay at 42°C followed by qPCR (Van Dyke 16S qPCR) was used for the detection of Campylobacter. Matrix spikes were again performed and showed that the assay was very sensitive for recovery of C. jejuni in an irrigation water matrix (Table 2), with as little as 2.6 CFU/300 ml being recovered. A total of six irrigation water samples were identified as containing Campylobacter (Fig. 3; Table 5) in 2015, with one sample containing two different species. Similar to the findings in 2014, the Campylobacter MPN was exceptionally low (1 to 2 Campylobacter/ 300 ml), depending upon the PCR assay used (Table 5). A. butzleri was again present in a large number of samples (54%), but the MPN was much lower than that in 2014 (13.5 versus 210/300 ml), likely due to the elevated incubation temperature of 42°C, which was not optimal for Arcobacter.

**TABLE 5:** Frequency and enumeration of Campylobacter and Arcobacter bacteria in irrigation water from 2015 as detected by a single-enrichment MPN-(q)PCR enrichment assay at 42°C (*n* = 74)

Bacterium and assay	No. (%) of bacteria detected	MPN (avg ± SD)/300 ml
Campylobacter		
Van Dyke 16S qPCR	6 (8.1)	1.0 ± 1.4
Jensen *glyA* qPCR	3 (4.1)	1.9 ± 2.1
Arcobacter hsp60 qPCR	40 (54.1)	13.5 ± 38.6

The Van Dyke 16S assay-positive MPN wells and isolates from BB agar were subsequently tested by four other PCR assays to determine their performance in detecting Campylobacter and in identifying it to the species level (Jensen *glyA*, Yamazaki multiplex PCR, Linton 16S, and Khan ITS assays; Table 1). The Linton 16S assay amplicons were sequenced from isolates, and it was determined that 5 of 6 samples contained C. jejuni or C. coli (the numbers of sequence polymorphisms in the Linton 16S assay amplicon were not sufficient to differentiate between C. jejuni and C. coli), while 2 of 6 samples contained C. lari (sample IW-6 contained multiple species) (Table 6). Interestingly, of the five C. jejuni/C. coli isolates, the Jensen *glyA*, Yamazaki multiplex PCR, and Khan ITS assays could correctly identify only one (IW-1) of these samples. The remaining four C. jejuni/C. coli isolates were identified only from Van Dyke 16S assay-positive samples by sequence analysis of the Linton *16S* assay amplicon. One of these samples (IW-4) was identified as C. coli by the Khan ITS assay (Table 6) but not by the Jensen *glyA* or Yamazaki multiplex assay. Another sample (IW-6) was identified as C. lari by the Jensen *glyA* assay but was negative by the Yamazaki assay and indeterminate by the Khan ITS assay in the MPN well. Multiple isolates from one sample (IW-6) were characterized, and both C. lari and C. jejuni/C. coli colonies were identified by sequence analysis of the Linton 16S assay amplicon, suggesting that multiple Campylobacter species were growing in the well. In addition, the lack of detection by the Jensen *glyA*, Yamazaki multiplex PCR, and Khan ITS assays suggests that the C. jejuni/C. coli bacteria growing in the sample IW-6 MPN well were similar to those of other irrigation water samples (IW-3, IW-5) for which none of these assays could identify Campylobacter in the MPN cultures but for which the Van Dyke 16S assay could detect these campylobacters (Table 6). The data suggest that the Van Dyke 16S assay is the most appropriate assay in terms of screening-level sensitivity and specificity for detection of environmental Campylobacter in water.

**TABLE 6:** Campylobacter PCR assay performance on Campylobacter enrichment cultures and isolates from irrigation water, wastewater, or human stool

Sample^*a*^	Detection by indicated assay (species detected)^*b*^					DNA sequence confirmation (16S gene) by Linton 16S endpoint PCR^*c*^
	Van Dyke 16S qPCR	Jensen *glyA* qPCR	Yamazaki multiplex PCR	Khan ITS multiplex PCR	Linton 16S endpoint PCR	
IW-1	+	+ (C. jejuni)^*d*^^,^^*e*^	+ (*C*. jejuni)	+ (C. jejuni)^*e*^	+	C. jejuni/C. coli^*e*^
IW-2	+	+ (C. lari)^*d*^^,^^*e*^	−	+ (*C*. lari)^*e*^	+	C. lari^*e*^
IW-3	+	−	−	−	+	C. jejuni/C. coli^*e*^
IW-4	+	−	−	+ (C. coli)^*b*^	+	C. jejuni/C. coli^*e*^
IW-5	+	−	−	−	+	C. jejuni/C. coli^*e*^
IW-6	+	+ (C. lari)^*d*^^,^^*e*^	−	+ (IND)^*d*^^,^^*e*^	+	C. jejuni/C. coli^*d*^^,^^*e*^, C. lari^*e*^
WW-1	+	+ (C. coli)	+ (C. coli)	+ (C. coli)	+	C. jejuni/C. coli^*e*^
WW-2	+	+ (C. coli)	+ (C. coli)	+ (C. coli)	+	C. jejuni/C. coli^*e*^
WW-3	+	+ (C. coli)	+ (C. coli)	+ (C. coli)	+	C. jejuni/C. coli^*e*^
WW-4	+	+ (C. jejuni)	+ (C. jejuni)	+ (IND)	+	C. jejuni/C. coli^*e*^
WW-5	+	+ (C. jejuni)	+ (C. jejuni)	+ (IND)	+	C. jejuni/C. coli^*e*^
WW-6	+	+ (C. jejuni)	+ (C. jejuni)	+ (C. jejuni)	+	C. jejuni/C. coli^*e*^
WW-7	+	+ (C. coli)	+ (C. coli)	+ (C. coli)	+	C. jejuni/C. coli^*e*^
PI-1	+	+ (C. coli)	+ (C. coli)	+ (C. coli)	+	C. jejuni/C. coli^*e*^
PI-2	+	+ (C. jejuni)	+ (C. jejuni)	+ (*C*. jejuni + C. lari)	+	C. jejuni/C. coli^*e*^
PI-3	+	+ (C. jejuni)	+ (jejuni)	+ (*C*. jejuni + C. lari)	+	C. jejuni/C. coli^*e*^
PI-4	+	+ (C. jejuni)	+ (jejuni)	+ (C. jejuni)	+	C. jejuni/C. coli^*e*^

a IW, irrigation water; WW, wastewater; PI, patient isolate.

b +, detected; −, not detected; IND, indeterminate.

c C. jejuni and C. coli cannot be distinguished from each other based on sequencing by the Linton 16S amplicon, whereas all other species of Campylobacter can be. The designation of C. jejuni/C. coli in this column reflects this.

d DNA amplified from MPN culture well.

e DNA amplified from isolate.

### Environmental C. jejuni/C. coli isolates are distinct from human isolates.

The majority of Campylobacter PCR assays reported in the literature have been developed using human Campylobacter isolates. Based on the inability of three different PCR assays (Jensen *glyA*, Yamazaki multiplex PCR, and Khan ITS assays) to detect 4 of 5 environmental C. jejuni/C. coli isolates, we decided to compare five different PCR assays for detecting campylobacters isolated from wastewater and human feces and identifying them to the species level. Seven Campylobacter isolates from wastewater and four isolates from human feces were also tested by the five PCR assays (Van Dyke 16S, Jensen *glyA*, Yamazaki multiplex, Khan ITS, and Linton 16S assays). For these isolates, 11 of 11 were detected by all of the PCR assays. The Jensen *glyA* qPCR and Yamazaki multiplex PCR assays produced 100% concordance and appeared to be accurate in identifying C. jejuni or C. coli of human origin but not of environmental origin (Table 6). There was, however, a lack of concordance with the results of the Khan ITS assay (Table 6). In particular, the Khan ITS assay often produced an indeterminate result due to abnormal band sizes or multiple bands (data not shown).

The results from comparing Campylobacter spp. isolated from different sources suggest that some environmental C. jejuni/C. coli isolates (samples IW-3 to IW-6) may be distinct from human isolates or type strains, with animals being the likely source. We were able to identify them as C. jejuni/C. coli by analysis of their 16S rRNA gene sequences. This observation suggests that current PCR methods for the identification of C. jejuni/C. coli from surface waters, which use PCR assays developed with patient isolates and/or type strains, are likely inadequate. The more inclusive and specific assay described by Van Dyke and colleagues (19) was the preferred detection/screening method, followed by 16S rRNA gene sequencing using a larger amplicon for better species resolution.

## DISCUSSION

Campylobacter is an enteric bacterium that can cause serious gastrointestinal illness in humans and more serious sequelae in a small percentage of cases. It is found in the gut of warm-blooded vertebrates such as birds, cattle, and pigs (reviewed in reference 37). Campylobacter is commonly found on food products due to fecal cross-contamination during slaughter. The identification of Campylobacter in water, however, is an indication of fecal contamination, as Campylobacter is not thought to grow in water due to its aerointolerance and specific growth needs. Instead, it adapts to these adverse conditions through various methods, including entry into viable-but-nonculturable (VNBC) states, formation of biofilms, or upregulation of oxidative stress response genes (reviewed in references 37 and 38). Fecal contamination contributing to Campylobacter deposition in water may come from human sewage discharge, overland runoff of feces from domestic farm animals, runoff from manure applied to fields, or direct deposition of feces from aquatic birds or mammals. The Campylobacter spp. from these sources are representative of the fecal input, as determined by multilocus sequence typing (39–41).

Several studies in Canada have reported high frequencies of detection of Campylobacter from surface waters (19, 21, 41–43), while others have reported detection of Campylobacter at levels up to 10^5^ MPN/100 ml near a wastewater discharge location (20). Ingestion of contaminated water can lead to outbreaks of campylobacteriosis, as the infectious dose of Campylobacter has been reported to be as low as 500 CFU (44). Estimating the concentration of Campylobacter in water is extremely important for microbial risk assessments associated with food (i.e., irrigation) and water (i.e., recreational water) exposures. The majority of studies looking at Campylobacter detection rates focus on qualitative presence/absence detection instead of quantitative methods, but qualitative methods provide little information about the public health safety of water use. In this study, we report the development of a quantitative assay for the detection and enumeration of Campylobacter spp. from water sources to help give context to the risk of contact with irrigation water (or wastewater) and its use on crops intended for consumption.

For the development of a quantitative method of detection of Campylobacter from water, the sample-processing step had to be considered, as large volumes of surface water are required for the procedure. The two logical choices were centrifugation and filtration. Filtration is generally incompatible with the MPN format, as cells must be removed from the filters in order to split the sample into a multivessel MPN format and there is no effective method for confirming removal of the cells from the filters. For the MPN format, centrifugation is the logical choice and has been shown to be effective in recovery of Campylobacter (26).

During development of the MPN-qPCR assay, it became clear that quantitative data interpretation could vary dramatically depending upon which methods (PCR, metabolic indicators [TTC], or growth-related turbidity in selective media) were used to “score” the MPN. At least two of the primer sets (Linton 16S and de Boer Lv1-16S) used in this and other studies clearly cross-reacted with Arcobacter. This led to the determination that Bolton broth medium readily supports the growth of the closely related genus Arcobacter, especially at temperatures below 42°C, and care must be taken not to misidentify Arcobacter as Campylobacter. This is crucial when attempting to score wells (positive/negative) on the MPN plates for subsequent enumeration of the Campylobacter spp. Culture turbidity or metabolic indicators are useful only if the medium is selective enough to allow growth of Campylobacter while suppressing growth of other nontarget genera. Our work with wastewater suggests that while culturing at 42°C will reduce Arcobacter levels ∼2 log_10_, there is still a population of A. butzleri that is thermotolerant and that could therefore confound molecular detection/confirmation. A previous report of a sequenced A. butzleri genome confirms that the A. butzleri genome appears to contain more antibiotic resistance genes than thermotolerant Campylobacter species (45), and we observed that the addition of two additional antibiotics to the culture did not substantively increase the selective power of the medium, respective to A. butzleri growth. In addition, the increased stringency associated with the additional two antibiotics lowered the level of Campylobacter detected by ∼40%. Based on these observations, a more specific method of scoring the MPN was required, as scoring the MPN by turbidity or metabolic indicators vastly overestimated the true levels of Campylobacter present. For this reason, we abandoned the double-enrichment method containing the metabolic indicator TTC used by Chenu et al. (28) and moved to a single-enrichment assay in Bolton broth with selective supplements at 42°C and used qPCR to score the MPN.

Using qPCR to score the MPN reduced the overall time and cost required to complete the assay, yet care must be taken to ensure the specificity of the PCR assays. Our testing of a variety of Campylobacter PCR assays demonstrated how paramount this decision is in data interpretation. The only assay that was fully inclusive of Campylobacter and exclusive of Arcobacter was the 16S rRNA gene assay developed by Van Dyke and colleagues (19), even though the assay itself had a higher LOD_95_ than other Campylobacter assays tested. The other molecular assays tested (i) cross-reacted with Arcobacter (Linton 16S and de Boer Lv1-16S assays), (ii) were unable to detect Campylobacter in a background of Arcobacter (de Boer LV1-16S assay), or (iii) missed a certain percentage of Campylobacter (Yamazaki multiplex, Khan ITS, and Jensen *glyA* assays) in the environment. PCR inclusivity is particularly important, as the Campylobacter spp. we isolated from irrigation water appeared to be distinct from the human and wastewater isolates that we tested. C. lari is a genetically diverse species, and the inability of the Yamazaki multiplex PCR to detect a subgroup of this species has been previously reported (34). The inability of C. jejuni- or C. coli-specific PCR assays (i.e., Khan ITS and Jensen *glyA* assays) to detect environmental isolates has not to our knowledge been reported before. In order to accurately quantitate Campylobacter spp. in irrigation water and to use this information for microbial risk assessments, the PCR assays must be as inclusive as possible of all Campylobacter spp. but exclusive of nontarget organisms (i.e., Arcobacter spp.), noting, however, that various Campylobacter species/strains from animals are less likely to be infectious in humans (46). Several reports of Campylobacter prevalence/concentration in water have relied on the Linton 16S primer set for some of their interpretations (20, 21, 43). We urge caution in the interpretation of the aforementioned results due to possible conflation with Arcobacter spp. in these studies. We suggest that the recommended procedure for Campylobacter detection in surface water should encompass the genus-specific 16S gene detection methods of Van Dyke et al. (19), followed by species identification of isolates by 16S gene sequencing and/or another genomic method such as comparative genomic fingerprinting, multilocus sequence typing (MLST), or *flaA* typing (methods reviewed in reference 47) for the resolution of C. coli and C. jejuni.

Limitations of culture-based assays to detect Campylobacter in surface waters include the findings that Campylobacter enters into a dormant or VNBC state in aquatic environments and if stored for a prolonged period at 4°C (48). Once in a VNBC state, recovery in a rich medium is often not possible (48). Hence, only relatively “fresh” campylobacters will be detected by culture-based methods. This, in combination with transportation time of samples to the laboratory, likely leads to an underestimation of the true viable Campylobacter numbers at the time of sampling. The use of propidium monoazide (PMA)-PCR has been reported for the direct detection by PCR of live (including VNBC) versus dead Campylobacter bacteria (49), but caution must be used, as uptake of this DNA intercalating dye that inhibits PCR has been shown to be inconsistent, affecting its efficacy (50, 51). Additionally, primer/probe selection is key to the accurate interpretation of any PMA assay due to the potential for nonspecific amplification of Arcobacter spp.

Arcobacter is an emerging pathogen, with several studies reporting it to be the fourth most common bacterial pathogen present (up to 1.3%) in human diarrhetic stools (52–54). Previous reports have shown Arcobacter butzleri to be present at high levels in wastewater (24, 55, 56), and our results confirm A. butzleri levels to be >10^5^ MPN/100 ml in raw wastewater in Alberta, Canada (J. Kim, G. Banting, B. Jeon, N. Ashbolt, and N. Neumann, unpublished data). Arcobacter has also been reported in fresh-vegetable processing plants (16) and fresh shellfish (57), both of which can be impacted by wastewater discharge. Based on our study of irrigation water in Alberta, Canada, Arcobacter butzleri was found at levels 2 to 3 log_10_ higher than Campylobacter spp. This observation, along with known environmental tolerance of Arcobacter (23), suggests that Arcobacter may represent a greater threat to human health than Campylobacter in the context of irrigation water. Hence, we feel that the detection and enumeration of Arcobacter levels in irrigation water (and other surface water) warrant further study. The described assay can easily be modified to quantitate Arcobacter bacteria by lowering the incubation temperature to 30°C, a more optimal growth temperature for this organism (58).

Our findings suggest that the reporting of Campylobacter levels in water is highly dependent upon the methods used and that great care must be used to ensure that Arcobacter is not being misidentified as Campylobacter. As a result of our comprehensive evaluation of both culture and molecular biology-based detection of Campylobacter spp. in water, we report that the prevalence of Campylobacter in irrigation water in Alberta is extremely low (2.5% in 2014 [2 of 80 samples] and 8% in 2015 [6 of 74 samples]), and even in cases where it is found in the water samples, the concentration of the bacteria is also low (<2 MPN/300 ml). In this context, Arcobacter spp. may represent a greater threat to human health than Campylobacter spp. from contact with irrigation water. The miniaturized MPN-qPCR assay described in this paper for estimating the occurrence of Campylobacter and Arcobacter in irrigation water and wastewater discharges should provide valuable input for the quantitative microbial risk assessment of water for which human contact or contaminated food consumption is likely.
